# Expression of miR-152 in cervical cancer and its influence on cisplatin resistance

**DOI:** 10.4314/ahs.v23i2.28

**Published:** 2023-06

**Authors:** Jun Wu, Xiaoping Chi, Qian Yang, Jinxiao Li

**Affiliations:** 1 Department of Obstetrics & Gynecology, The First People's Hospital of Wenling, Wenling 317500, Zhejiang Province, China; 2 Wenzhou Medical University, Affiliated Wenling Hospital, Wenling 317500, Zhejiang Province, China; 3 Department of Radiology, The First People's Hospital of Wenling, Wenling 317500, Zhejiang Province, China

**Keywords:** miR-152, cervical cancer, cisplatin, drug resistance

## Abstract

**Background:**

Cervical cancer has a high mortality rate.

**Aim:**

We aimed to study the expression of micro ribonucleic acid 152 (miR-152) in cervical cancer and its influence on cisplatin (DDP) resistance.

**Methodology:**

Cervical cancer Hela cells were divided into control, DDP, DDP + mimic nc and DDP + miR-152 mimic groups.

**Results:**

DDP, DDP + mimic nc and DDP + miR-152 mimic groups had lower cell survival rate, smaller number of single clones and cells penetrating the membrane, and higher apoptosis rate and miR-152 expression than those of the control group (P<0.05). Compared with DDP and DDP + mimic nc groups, the cell survival rate, number of single clones and number of cells penetrating the membrane significantly decreased, while the apoptosis rate and miR-152 expression increased in the DDP + miR-152 mimic group (P<0.05). ERBB3 was a downstream target gene of miR-152. Hela cells transfected with miR-152 mimic had lower protein expressions of Snail, ERBB3, Akt2, p-Akt and c-myc than those of NC cells (P<0.05).

**Conclusion:**

MiR-152 suppresses the proliferation, migration and infiltration of cervical cancer cells and reduces their resistance to DDP chemotherapy by inhibiting the expressions of proteins in the *ERBB3/Akt/c-myc* and *ERBB3/Akt/Snail* pathways.

## Introduction

Cervical cancer, as a kind of tumor of the female reproductive system, has a high mortality rate. In clinical practice, middle-advanced stage cervical cancer is mainly treated through radiotherapy and chemotherapy, and cisplatin (DDP) is a chemotherapeutic drug commonly used to treat cervical cancer. Different patients with cervical cancer have various sensitivities to chemotherapy with DDP, and some are resistant to DDP, affecting the treatment outcomes. Micro ribonucleic acids (miRNAs) play key roles in the occurrence, development, invasion, and treatment of tumors^1^. Among them, miR-152 wors as a tumor suppressor gene in many cancers such as ovarian cancer, liver cancer and colorectal cancer^2^. MiR-152 has a down-regulated expression in colorectal cancer tissues and cells, and can inhibit the proliferation and migration and facilitate the apoptosis of colorectal cancer cells^3^. However, the expression of miR-152 in cervical cancer or its effect on the DDP resistance of cervical cancer cells has seldom been referred. In this study, cervical cancer Hela cell line was used. MiR-152 was overexpressed to measure its expression in cervical cancer, and its influence on the DDP resistance of cervical cancer cells was assessed, aiming to provide novel insights into clinical treatment.

## Materials and methods

### Baseline clinical data

A total of 124 patients who were pathologically diagnosed as cervical cancer and received surgical treatment in our hospital from June 2018 to August 2019 were selected. They were aged 42-76 years old, with an average of (57.42±16.7) years old. The course of disease was 8 months to 4 years. Among the 124 patients, 72 were post-menopausal patients, with a mean menopausal duration of 7 years. All patients underwent routine urine examination, cervical histopathological examination, routine blood examination, biochemical examination and other auxiliary examinations. Then the TNM staging system was employed for pathological staging (AJCC cancer staging manual). All patients met the 2012 National Comprehensive Cancer Network (NCCN) diagnostic criteria for cervical cancer^4^. The surgically resected specimens were subjected to determination of clinicopathological characteristics and pathological examination. Besides, cervical tissues more than 3 cm away from the cervical cancer tissues were collected as adjacent tissues. All patients have signed the consent form.

### Materials

Cervical cancer Hela cells and Hela/DDP cells was obtained from the Laboratory of Obstetrics and Gynecology in Wuhan Union Hospital (China). Acridine orange and DDP freeze-dried powders were bought from Beyotime Biotechnology Co., Ltd. and Qilu Pharmaceutical Co, Ltd. (China), respectively. Dulbecco's modified Eagle medium (DMEM), TRIzol reagent and Lipofectamine2000 were obtained from Invitrogen (USA). Transcriptor first strand complementary deoxyribonucleic acid (cDNA) synthesis kit, real-time polymerase chain reaction (RT-PCR) kit, One Step Prime Script miRNA cDNA synthesis kit and SYBR Premix Ex Taq II were provided by TaKaRa (Japan). Real-time fluorescence quantitative amplification system was purchased from Applied Biosystems (USA). NanoDrop2000c protein nucleic acid detector was provided by Thermo (USA). Dimethyl sulfoxide (99.9%) was obtained from Sigma (USA). Fetal bovine serum (FBS) was bought from Procell Life Science and Technology Co, Ltd. (China). Fluorescein isothiocyanate cell apoptosis detection kit was provided by Nanjing Bio box Biotech. Co., Ltd. (China). The specific primers for miR-152, miR-152 mimic and internal reference U6 were all purchased from Qiagen (Germany).

### Detection of miR-152 expressions in cervical cancer and adjacent tissues by qRT-PCR

Total RNAs were extracted from adjacent tissues and cervical cancer tissues using TRIzol reagents and reversely transcribed into cDNAs using One Step Prime Script miRNA cDNA synthesis kit. Next, real-time qPCR was performed using SYBR Premix Ex Taq II. Endogenous U6 small nuclear RNA (U6) was utilized for normalization (U6 forward primer: 5′-TAGGTCCAACCTTATA-3′, reverse primer: 5′-CCGCGAAGTGCTTAAACGCT-3′). The 2^-ΔΔCT^ method was used to calculate the relative expression level of miR-152. The sequences of PCR primers were as follows: miR-152 forward primer: 5′-TCAGATATGTTCCCGTTCGAGAG-3′, and reverse primer: 5′-GGGTTATGCTGGTTTAGGCAA-3′. The reaction system consisted of 10 µL of reverse primer, 10 µL of forward primer, 0.6 µL of RNA template, 0.2 µL of super enzyme mixture, 5 µL of qRT-PCR buffer and 3.4 µL of RNA-free water. The cycle process was maintained at 95°C for 10 min, 40 cycles, and at 60°C for 15 s.

### Cell culture and transfection

Hela cells was added to RPMI 1640 containing 1% penicillin-streptomycin, and cultured in an incubator with 5% CO2 and 20% O2 at 37°C. Then the cells was digested and passaged with 0.5% trypsin upon reaching 80% confluence. Hela/DDP cell line was subjected to culture under the same conditions.

A total of 10 mL of Hela/DDP cell solution was inoculated into a 6-well plate. Meanwhile, 200 µL of DMEM was added 100 pmol of miR-152 mimic and then 4 µL of Lipofectamine 2000, mixed and cultured at room temperature for 30 min. The mixture was added to the 6-well plate to wash cells, the solution was discarded, and DMEM was added, followed by 24 h of culture. Afterwards, the transfection efficiency was measured by flow cytometry. The same treatment was conducted for mimic nc.

### Experimental grouping

Four groups were set, including control group [10 mL of (5×106) Hela cell solution was added to DMEM containing 10% FBS and cultured in the incubator with 5% CO_2_ and 20% O_2_ at 37°C], DDP group [10 mL (5×106) Hela/ DDP cell solution was added to DMEM containing 10% FBS and cultured in the incubator with 5% CO_2_ and 20% O_2_ at 37°C], DDP + miR-152 mimic group [10 mL of Hela/DDP cell solution transfected with miR-152 mimic was added with 5 mL of DDP (5 µmol/L) and cultured in the incubator with 5% CO_2_ and 20% O_2_ at 37°C], and DDP + mimic nc group [10 mL of Hela/DDP cell solution transfected with mimic nc was added with 5 mL of DDP (5 µmol/L) and cultured in the incubator with 5% CO_2_ and 20% O_2_ at 37°C]. Six parallel samples were set for each well, and the culture time was 72 h.

### Detection of cell survival rate and number of single clones

The above 4 groups of cells was inoculated into 6-well plate, added 10 µL of 5 mg/mL methyl thiazolyl tetrazolium (MTT) solution to each well and cultured in the incubator at 37°C for 4 h. Then the optical density (OD) value at the wavelength of 490 nm was read using a microplate reader.

After being cultured in DMEM containing 10% FBS for 5 d, the cells were stained with methylene blue, and an inverted microscope (×400) was used to count the colonies formed in the imaging area.

### Detection of cell apoptosis by flow cytometry

The cells cultured for 72 h were trypsin zed and then stained with Annexin V-FITC and PI solution for 15 min in dark. Afterwards, the apoptosis rate was detected using a flow cytometer.

### Detection of cell migration by Transwell assay

A 2 mm-thick Matrigel gel was evenly coated on the polycarbonate microporous filter membrane. Then 60 µL of cell solution taken from each group was added to the chamber, and DMEM containing FBS was added, followed by 12 h of culture at room temperature. The cells in the filter chamber were gently collected using sterile medical cotton swabs, fixed and stained. Afterwards, 5 visual fields (center and surrounding fields) at high power were selected, and the number of cells penetrating the 8 µm micropore in each visual field was recorded.

### Detection of miR-152 mRNA expression in cells by qRT-PCR

The cell solution (5 mL, 5×10^6^ cells/mL) was collected, centrifuged at 5000 rpm for 5 min, and frozen at -70°C for later use. The expression level of miR-152 was measured by qRT-PCR.

### Western blotting

The collected cells were added RIPA cell lysis buffer, followed by ice bath for 10 min. Then the protein concentration was determined by BCA assay. Sodium dodecyl sulfate (SDS) loading buffer was added and subjected to water bath at 95°C for 10 min for protein denaturation. The protein bands were separated by 10% SDS-polyacrylamide gel electrophoresis and transferred to a PVDF membrane through semi-dry transfer method. Then the membrane was incubated with antibodies against *ERBB3* (diluted at 1:500), *c-myc* (diluted at 1:500), *p-Akt* (diluted at 1:1000), and Snail (diluted at 1:1000) added in order on a shaker at 4°C overnight. Afterwards, they were incubated with secondary antibody (diluted at 1:5000) at 37°C for 1 h, followed by 5 min of development with an appropriate amount of ECL reagent. Next, the OD value was measured by a gel imaging analysis system (Alpha, USA).

### Dual-luciferase reporter gene assay

The possible targets of miR-152 were predicted using the online prediction tools including microrna, miRanda and Target Scan. It was confirmed through blast alignment that *ERBB3* was a target gene of miR-152, and the 3′ untranslated region (3′-UTR) sequence of *ERBB3* interacted with the sequence of miR-152. Luciferase reporter plasmids were constructed by separately inserting the 3′-UTR sequences of wild-type *ERBB3* and deletion mutant *ERBB3* into the XbaI and FseI sites of the pGL3 luciferase reporter vector for PCR identification.

Human epithelial cells HEK293T (Institute of Biochemistry and Cell Biology, Shanghai Institutes for Biological Sciences, Chinese Academy of Sciences) were inoculated into the 6-well plate and transfected with miR-152 mimics according to the instructions of liposome transfection. The luciferase activity was detected using GloMax-TM 20/20 luminometer (Promega, USA).

### Statistical analysis

GraphPad Prim 5.01 software and SPSS 19.0 software were employed for plotting and statistical analysis, respectively. One-way ANOVA was used for comparisons among groups, and the t test was employed for comparisons between two groups. P<0.05 suggested that a difference was statistically significant.

## Results

### Differential expressions of miR-152 in cervical cancer and normal adjacent tissues

**Figure 1:**
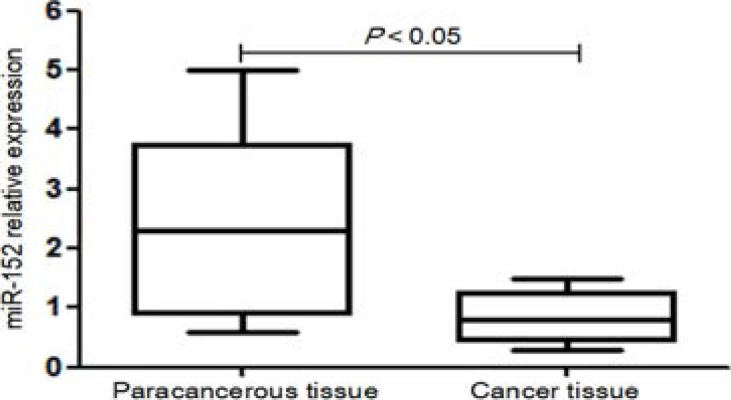
Differential expressions of miR-152 in cervical cancer and normal adjacent tissues

The miR-152 expression in cervical cancer tissues was significantly lower than that in adjacent tissues (P<0.01) (Figure 1).

### MiR-152 expression levels in different groups of cells

**Figure 2:**
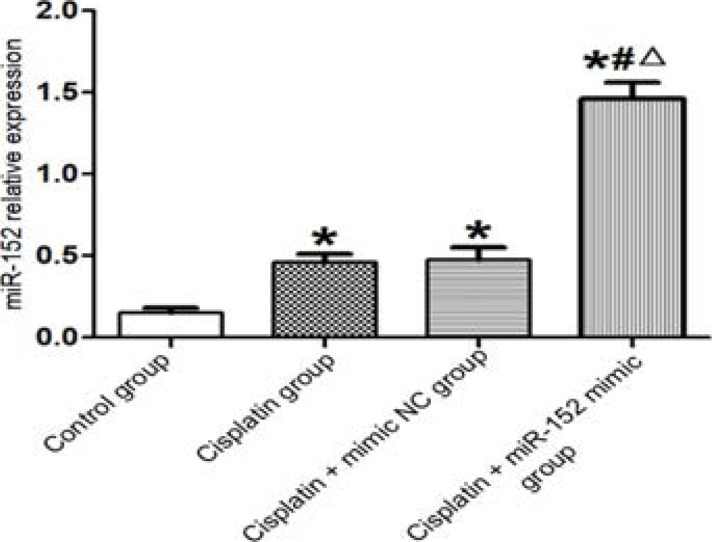
MiR-152 expression levels in different groups of cells. *P<0.01 vs. control group, #P<0.01 vs. DDP group, ΔP<0.01 vs. DDP + mimic nc group

Based on the results of qRT-PCR, the expressions of miR-152 in DDP, DDP + mimic nc and DDP + miR-152 mimic groups were significantly higher than that in the control group, and the expressions in DDP and DDP + mimic nc groups were significantly lower than that in the DDP + miR-152 mimic group (P<0.05). DDP and DDP + mimic nc groups had similar miR-152 expressions (P>0.05) (Figure 2). Therefore, miR-152 was successfully transfected, and high miR-152 expression could be induced by DDP.

### Effect of miR-152 on cell proliferation

**Figure 3:**
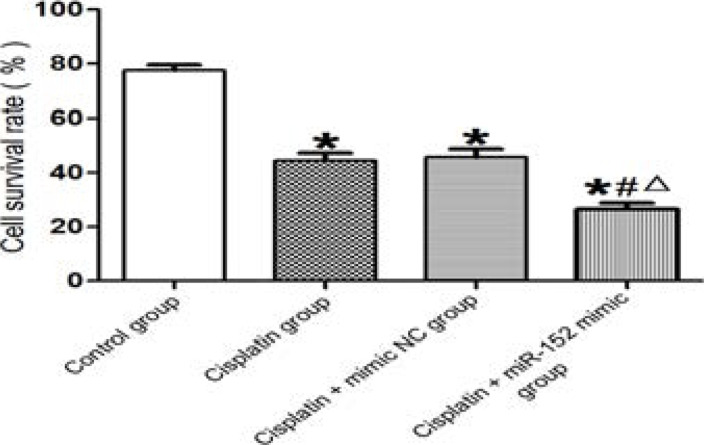
Effect of miR-152 on cell proliferation *P<0.01 vs. control group, #P<0.01 vs. DDP group, ΔP<0.01 vs. DDP + mimic nc group

The MTT assay results showed that compared with the control group, the survival rates of cells in DDP, DDP + mimic nc and DDP + miR-152 mimic groups were significantly lower (P<0.05). In comparison with the DDP + miR-152 mimic group, DDP and DDP + mimic nc groups had significantly higher cell survival rates (P<0.05). DDP and DDP + mimic nc groups had similar survival rates (P>0.05) (Figure 3). Thus, miR-152 was able to suppress the proliferation of Hela cells.

### Effect of miR-152 on number of single clones of cells

**Figure 4:**
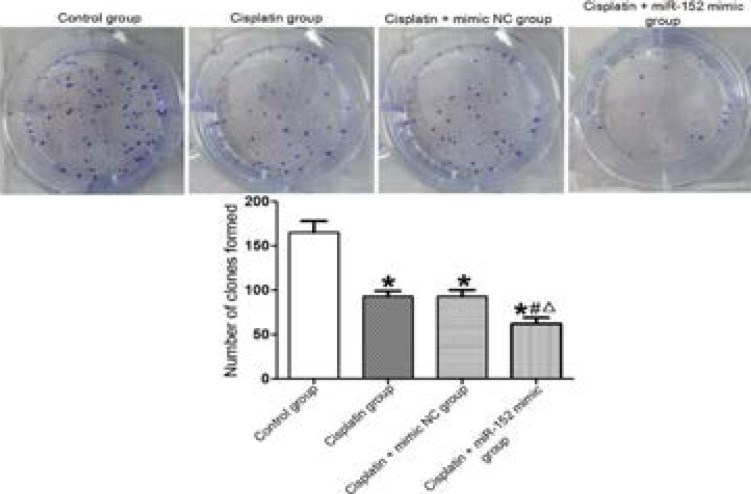
Effect of miR-152 on number of single clones of cells. *P<0.01 vs. control group, #P<0.01 vs. DDP group, ΔP<0.01 vs. DDP + mimic nc group

The MTT assay results exhibited that the numbers of single clones in DDP, DDP + mimic nc and DDP + miR-152 mimic groups were significantly smaller than that of the control group, and the numbers of DDP and DDP + mimic nc groups were significantly larger than that of the DDP + miR-152 mimic group (P<0.05). DDP and DDP + mimic nc groups had similar numbers of single clones (P>0.05) (Figure 4).

### Effect of miR-152 on cell apoptosis rate

**Figure 5:**
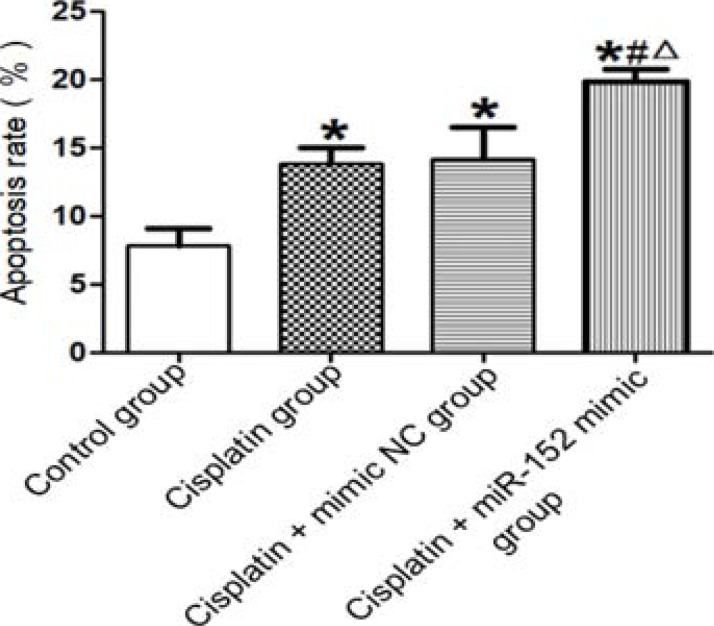
Effect of miR-152 on cell apoptosis rate. *P<0.01 vs. control group, #P<0.01 vs. DDP group, ΔP<0.01 vs. DDP + mimic nc group

It was found though flow cytometry that the apoptosis rate was significantly elevated in DDP, DDP + mimic nc and DDP + miR-152 mimic groups compared with that of the control group, while significantly lowered in DDP and DDP + mimic nc groups compared with that of the DDP + miR-152 mimic group (P<0.05). DDP and DDP + mimic nc groups had similar apoptosis rates (P>0.05) (Figure 5).

### Effect of miR-152 on number of cells penetrating the membrane

**Figure 6:**
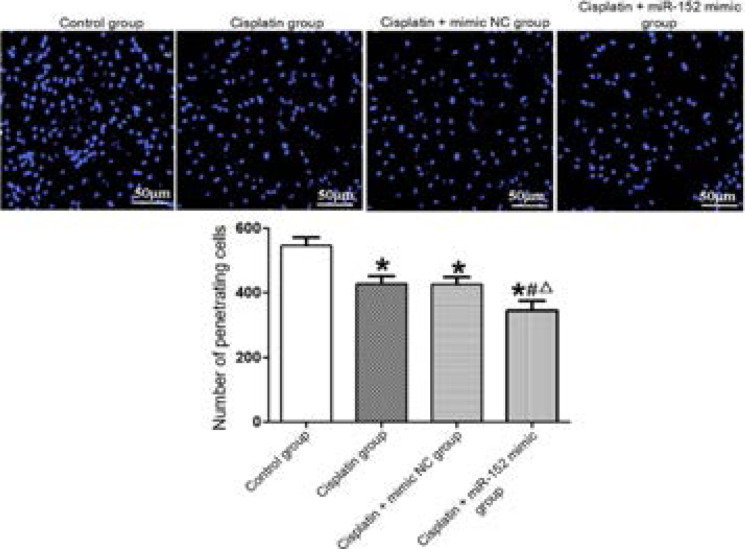
Effect of miR-152 on number of cells penetrating the membrane (×400). *P<0.01 vs. control group, #P<0.01 vs. DDP group, ΔP<0.01 vs. DDP +

In accordance with the results of Transwell migration assay, the number of cells penetrating the membrane was significantly smaller in DDP, DDP + mimic nc and DDP + miR-152 mimic groups than that of the control group, and significantly larger in DDP and DDP + mimic nc groups than that of the DDP + miR-152 mimic group (P<0.05). DDP and DDP + mimic nc groups had similar numbers of cells penetrating the membrane (P>0.05) (Figure 6).

**Figure 7:**
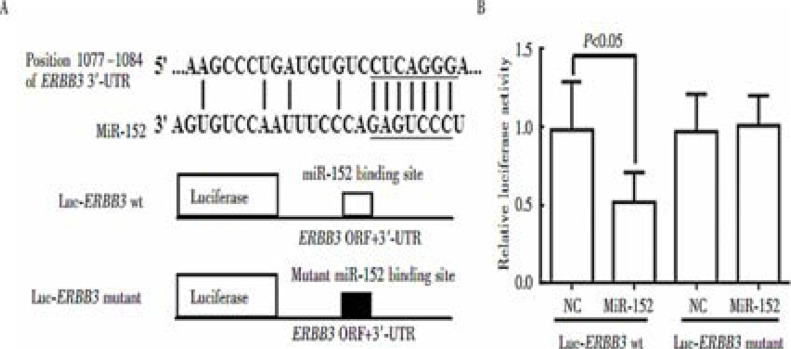
Dual-luciferase gene reporter assay results. A: Targets of miR-152 in the ERBB3 sequence and mutation sites of ERBB3 predicted through Target Scan, B: Activity of ERBB3 in Hela cells transfected with wild-type or mutant ERBB3 and miR-152 mimic detected by luciferase reporter gene assay (P<0.05)

ERBB3 was finally chosen as the potential target gene for subsequent experiments based on the possible downstream target genes of miR-152 predicted using the target gene prediction databases Target Scan, microrna and miRanda. Then the luciferase reporter gene plasmids containing the target gene wild-type or mutant 3′-UTR were constructed to determine the inhibitory effect of endogenous miR-152. The luciferase activity was significantly weakened (t=3.158, P=0.004) after transfection of miR-152 mimics into the gene reporter plasmid with cloned wild-type ERBB3 3′-UTR for 48 h, while hardly changed after transfection into the gene reporter plasmid with mutant 3′-UTR gene for 48 h. Hence, ERBB3 may be the downstream target gene of miR-152 (Figure 7).

**Figure 8:**
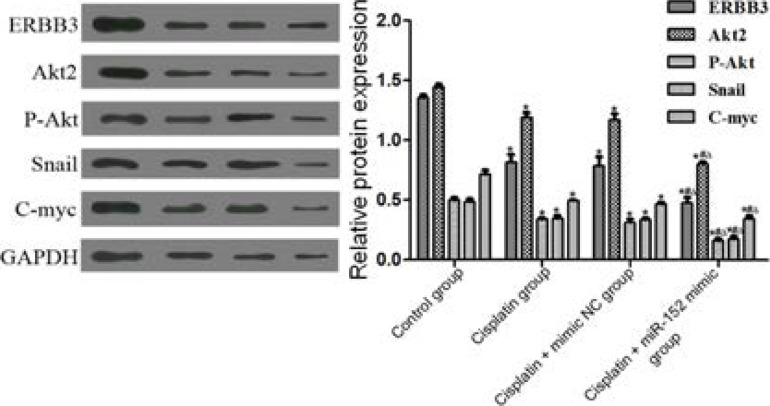
Targeting effect of miR-152 on proteins ERBB3, Akt2, p-Akt, Snail and c-myc. *P<0.01 vs. control group, #P<0.01 vs. DDP group, ΔP<0.01 vs. DDP + mimic nc group

Effect of up-regulation of miR-152 expression on expressions of proteins in the ERBB3/Akt/c-myc pathway The results of Western blotting showed that the protein expression levels of ERBB3, Akt2, p-Akt, Snail and c-myc in cells significantly reduced in DDP, DDP + mimic nc and DDP + miR-152 mimic groups compared with those in the control group, while significantly rose in DDP and DDP + mimic nc groups compared with those in the DDP + miR-152 mimic group (P<0.05). DDP and DDP + mimic nc groups had similar protein expression levels of ERBB3, Akt2, p-Akt, Snail and c-myc in cells (P>0.05) (Figure 8).

## Discussion

The incidence rate of cervical cancer in developing countries is about 10 times that in developed countries. In China, the incidence rate of cervical cancer ranks first among those of all gynecological malignancies^5^, and the mortality rate of cervical cancer in rural areas is 48% higher than that in urban areas^6^. In clinical practice, cervical cancer is mainly treated by surgery, radiotherapy, chemotherapy and their combination. As the first-choice drug for tumor chemotherapy, DDP exerts obvious antitumor effects. However, the therapeutic effect of DDP is affected by the drug resistance of tumor cells, as a crucial factor for recurrence. The DDP resistance is caused by increased DNA damage repair ability, inhibited mismatch repair, enhanced rejection of cells to drugs, decreased drug intake by individual cells and blocked apoptosis pathways^7^. MiRNAs are non-coding RNA molecules widely existing in eukaryotic cells, with about 22-24 nucleotides in length. The expressions of miRNAs have been closely associated with the occurrence and development of various tumors^8^. MiR-152, as a member of the miR-148/miR-152 family, is located on the chromosome 17, which functions as a tumor suppressor gene in many cancers including endometrial cancer, breast cancer and ovarian cancer^9^. MiR-152 has a lower expression level in cervical cancer tissues than that in adjacent tissues^10^, being in line with the results of this study.

In this study, the expression level of miR-152 in Hela cells treated with DDP was significantly higher than that in the cells not treated with DDP, demonstrating that DDP induced high miR-152 expression. The cancer cells treated with DDP and transfected with miR-152 had a significantly higher expression level of miR-152 than that of the cells only treated with DDP, indicating that miR-152 was successfully transfected. Besides, the cells treated with DDP exhibited a significantly lower survival rate, a smaller number of single clones and cells penetrating the membrane and a higher apoptosis rate than those of the cells not treated with DDP. The survival rate of cells, number of single clones and number of cells penetrating the membrane significantly decreased in the cells treated with DDP and transfected with miR-152 compared with those of the cells not treated with DDP, while the apoptosis rate was significantly higher in the former cells. Similar to a previous literature^11^, DDP affected the biological function of cancer cells by modulating the expression of miR-152, thus affecting their drug resistance.

MiRNAs can bind the downstream target gene 3′-UTR, 5′-UTR or CDS region for sequence complementation and binding. To determine the way by which miRNAs bind target genes, the miRNA regulatory network was investigated in this study using the online miRNA target gene prediction systems miRanda, microrna and Target Scan, showing that *ERBB3* may be a downstream target gene of miR-152. *ERBB3* was selected as the downstream target gene of miR-152 for further mechanism analysis in accordance with the thermal stability during the formation of dimers and sequence complementary pairing and stability of the corresponding secondary structure. It is well known that epithelial-mesenchymal transition (EMT), a highly conservative biological process, plays a key player role in the proliferation, differentiation, invasion and metastasis of tumor cells^12^. Besides, EMT affects the expressions of Snail and other transcription factors through many signalling pathways^13^. *C-myc* is a proto-oncogene enable the indefinite proliferation of cells, and its excessive or inappropriate production is a marker of many cancers. *P-Akt* is capable of increasing the transcriptional activity of *Snail* and *c-myc^14^. ERBB3*, a vital member of the ERBB family, is able to activate the *PI3K*/Akt pathway by binding the p85 subunit of *PI3K^15^*. In this study, the expressions of *ERBB3, Akt2,p-Akt, Snail* and *c-myc* significantly reduced in Hela cells treated with DDP, and the relative protein expression levels were significantly lower in cells treated with DDP and transfected with miR-152 mimic than those in the cells only treated with DDP. Accordingly, DDP induced miR-152 expression to significantly reduce the expression of ERBB3 and to inhibit the expressions of related genes of the *ERBB3/Akt/c-myc* and *ERBB3/Akt/Snail* pathways, thereby expediting the biological processes (such as proliferation and differentiation) of tumors.

In conclusion, miR-152 is lowly expressed in cervical cancer tissues, and its overexpression suppresses the proliferation, migration and infiltration and reduces the DDP resistance of cervical cancer cells by inhibiting the expressions of related proteins of the ERBB3/Akt/c-myc and ERBB3/Akt/Snail pathways and EMT. MiR-152 is expected to be a potential therapeutic target for cervical cancer to accelerate the research and development of targeted drugs.
